# Genetic Analysis of the CDI Pathway from *Burkholderia pseudomallei* 1026b

**DOI:** 10.1371/journal.pone.0120265

**Published:** 2015-03-18

**Authors:** Sanna Koskiniemi, Fernando Garza-Sánchez, Natasha Edman, Swarnava Chaudhuri, Stephen J. Poole, Colin Manoil, Christopher S. Hayes, David A. Low

**Affiliations:** 1 Department of Molecular, Cellular and Developmental Biology, University of California Santa Barbara, Santa Barbara, California, United States of America; 2 Biomolecular Science and Engineering Program, University of California Santa Barbara, Santa Barbara, California, United States of America; 3 Department of Genome Sciences, Box 355065, University of Washington, Seattle, Washington, United States of America; Centre National de la Recherche Scientifique, Aix-Marseille Université, FRANCE

## Abstract

Contact-dependent growth inhibition (CDI) is a mode of inter-bacterial competition mediated by the CdiB/CdiA family of two-partner secretion systems. CdiA binds to receptors on susceptible target bacteria, then delivers a toxin domain derived from its C-terminus. Studies with *Escherichia coli* suggest the existence of multiple CDI growth-inhibition pathways, whereby different systems exploit distinct target-cell proteins to deliver and activate toxins. Here, we explore the CDI pathway in *Burkholderia* using the CDI_II_^Bp1026b^ system encoded on chromosome II of *Burkholderia pseudomallei* 1026b as a model. We took a genetic approach and selected *Burkholderia thailandensis* E264 mutants that are resistant to growth inhibition by CDI_II_^Bp1026b^. We identified mutations in three genes, BTH_I0359, BTH_II0599, and BTH_I0986, each of which confers resistance to CDI_II_^Bp1026b^. BTH_I0359 encodes a small peptide of unknown function, whereas BTH_II0599 encodes a predicted inner membrane transport protein of the major facilitator superfamily. The inner membrane localization of BTH_II0599 suggests that it may facilitate translocation of CdiA-CT_II_^Bp1026b^ toxin from the periplasm into the cytoplasm of target cells. BTH_I0986 encodes a putative transglycosylase involved in lipopolysaccharide (LPS) synthesis. ∆BTH_I0986 mutants have altered LPS structure and do not interact with CDI^+^ inhibitor cells to the same extent as BTH_I0986^+^ cells, suggesting that LPS could function as a receptor for CdiA_II_^Bp1026b^. Although ∆BTH_I0359, ∆BTH_II0599, and ∆BTH_I0986 mutations confer resistance to CDI_II_^Bp1026b^, they provide no protection against the CDI^E264^ system deployed by *B*. *thailandensis* E264. Together, these findings demonstrate that CDI growth-inhibition pathways are distinct and can differ significantly even between closely related species.

## Introduction

Contact-dependent growth inhibition (CDI) is a mechanism of inter-cellular competition used by some Gram-negative species to inhibit the growth of neighboring bacteria [1–3]. CDI is mediated by the CdiB/CdiA family of two-partner secretion proteins, which are distributed through α-, β- and γ-proteobacteria [4]. CdiB is an outer-membrane β-barrel protein that exports the CdiA toxic effector. CdiA proteins are very large (180–650 kDa depending on the species) and are predicted to form long β-helical filaments that extend from the surface of inhibitor cells [2,5]. During CDI, CdiA binds to specific receptors on susceptible bacteria and delivers a toxin domain derived from its C-terminal region (CdiA-CT). CdiA-CT sequences are highly variable between bacterial species and strains, but the N-terminal boundary of this region is typically delineated by a highly conserved VENN peptide motif [1,6]. CdiA-CT sequence diversity suggests a variety of toxin activities, and indeed most characterized CDI toxins are nucleases with different cleavage specificities for DNA, tRNA or rRNA [1,7–9]. Additionally, CdiA-CT^EC93^ from *Escherichia coli* EC93 appears to form pores in target-cell membranes [10], and sequence analysis suggests that other CDI toxins may have RNA deaminase and protease/peptidase activities [11]. CDI^+^ bacteria protect themselves from auto-inhibition by producing CdiI immunity proteins, which bind to CdiA-CT toxins and neutralize their activities.

CDI has been characterized most extensively in γ-proteobacteria, with *E*. *coli* EC93 and uropathogenic *E*. *coli* 536 (UPEC 536) serving as model systems. Studies with those systems have revealed that CDI exploits specific target-cell proteins to deliver growth inhibitory toxins [12,13]. Selections for mutants that are resistant to the *E*. *coli* EC93 system (CDI^EC93^) identified *bamA* and *acrB* mutations that protect target cells from growth inhibition [12]. BamA is an essential outer-membrane protein required for the assembly of all β-barrel proteins [14–17], and is specifically recognized as a target-cell receptor by CdiA^EC93^ [12,18]. AcrB is a trimeric integral membrane protein that functions together with AcrA and TolC as a multi-drug efflux pump [19]. However, the efflux function of AcrB is not required for CDI^EC93^ because Δ*acrA* and Δ*tolC* mutants are both fully sensitive to CDI^EC93^ [12]. Though the role of AcrB during CDI^EC93^ is not known, its localization suggests that it could facilitate assembly of the CdiA-CT^EC93^ pore-forming toxin into the target-cell inner membrane. Biochemical studies on CdiA-CT^536^ from UPEC 536 have shown that this toxin is a latent tRNase that only exhibits nuclease activity when bound to the cysteine synthase, CysK [13]. In accord with *in vitro* studies, *E*. *coli* Δ*cysK* mutants are completely resistant to inhibition by CDI^UPEC536^. Collectively, these findings indicate that CDI pathways can encompass at least three distinct steps: i) receptor-binding to identify target bacteria, ii) translocation of CdiA-CT toxin across the target-cell envelope, and iii) activation of the toxin in the target-cell cytoplasm. Notably, the protective effects of *cysK* and *acrB* mutations are specific to the CDI^UPEC536^ and CDI^EC93^ pathways, respectively [13]. These findings raise the possibility that each CDI system/toxin exploits a unique set of proteins to inhibit target-cell growth.

CdiB and CdiA share significant homology across the proteobacteria, but the CDI systems of Burkholderiales exhibit a number of differences compared to other bacteria. Firstly, the variable toxin region in *Burkholderia* CdiA is typically demarcated by the (E/Q)LYN peptide motif rather than the VENN sequence found in most other bacteria [9,20]. *Burkholderia* toxins are modular and can be exchanged readily between *Burkholderia* CdiA proteins [9], but chimeric *E*. *coli* CdiA proteins carrying *Burkholderia* CdiA-CTs fused at the VENN sequence are not functional in CDI [1]. Secondly, CDI genes are arranged as *cdiAIB* clusters in *Burkholderia*, *Variovorax* and *Cupriavidus* species rather than the *cdiBAI* order found in other bacteria. This alternative gene arrangement is also correlated with a lack of "orphan" *cdiA-CT/cdiI* gene pairs. Orphan modules resemble the displaced 3´-fragments of full-length *cdiA* genes together with their cognate *cdiI* immunity genes [3,21]. Tandem arrays of orphan *cdiA-CT/cdiI* gene pairs are commonly found downstream of *cdiBAI* loci in γ-proteobacteria, and all strains of *Neisseria meningitidis* also carry well-defined orphan toxin/immunity clusters [21,22]. Finally, many *Burkholderia* CDI systems encode a small predicted lipoprotein, BcpO, between the *cdiI* and *cdiB* genes [20]. The function of BcpO is not understood completely, but it is required for CdiA secretion in *Burkholderia thailandensis* E264 [20]. Collectively, these observations suggest that the mechanisms of CDI in *Burkholderia* species are fundamentally distinct from other bacteria.

Here, we begin exploring *Burkholderia* CDI pathways using the CDI_II_^Bp1026b^ system encoded on chromosome II of *Burkholderia pseudomallei* 1026b as a model. We took a genetic approach and isolated transposon mutants of *B*. *thailandensis* E264 that are resistant to inhibition by the CDI_II_^Bp1026b^ system. Independent selections identified multiple transposon insertions in three genes—BTH_I0359, BTH_II0599, and BTH_I0986, each of which confers resistance to CDI_II_^Bp1026b^. BTH_I0359 encodes a small cytosolic protein of unknown function, BTH_II0599 encodes an integral membrane protein from the major facilitator superfamily (MFS), and BTH_I0986 encodes a predicted lipopolysaccharide (LPS) transglycosylase. We find that LPS structure is altered in BTH_I0986 mutants, suggesting that LPS may function as a receptor or co-receptor for CdiA_II_^Bp1026b^. These results demonstrate that the CDI_II_^Bp1026b^ is distinct from previously described *E*. *coli* pathways, suggesting that multiple pathways exist to translocate CDI toxins into target bacteria.

## Materials and Methods

### Bacterial strains and growth conditions

Bacterial strains were derived from *Burkholderia thailandensis* E264 and are listed in Table 1. Bacteria were routinely cultured in LB media supplemented with the following antibiotics where appropriate: kanamycin (Kan) 500 μg/mL; tetracycline (Tet) 25 μg/mL; trimethoprim (Tp) 100 μg/mL; chloramphenicol (Cam) 34 μg/mL; and polymyxin B (PB) 100 μg/mL. CDI_II_^Bp1026b^ competitions used Bt81 inhibitors, which are *B*. *thailandensis* E264 cells that express *cdiAIB*_II_^Bp1026b^ from plasmid pJSW1–6 (Table 2) [9]. Bt81 inhibitors and target cells were grown individually for at least 48 h (to OD_600_ > 0.6) in M9-minimal media supplemented with 0.2% L-arabinose. Approximately 10^9^ colony-forming units (cfu) of Bt81 inhibitors and 10^8^ cfu of target cells were mixed in 150 μL of M9-minimal medium supplemented with 0.2% arabinose, 1 μg/mL thiamine and 0.3 μg/mL ferric citrate, and aliquots plated onto LB agar supplemented with Tet or Kan to enumerate viable inhibitors and targets (respectively) at time 0 h. The remaining cell mixture (100 μL) was spread onto M9-minimal medium agar supplemented with 0.2% L-arabinose, 1 μg/mL thiamine and 0.3 μg/mL ferric citrate and incubated for 24 h at 30°C. Cells were then harvested from the agar surface, and viable inhibitor and target cell counts were determined as total cfu on Tet and Kan (respectively) supplemented LB agar. The competitive index (C.I.) was calculated as the ratio of target cells to inhibitor cells at 24 h divided by the target to inhibitor ratio at time 0 h. CDI^E264^ competitions were conducted in a similar manner, except inhibitor and target cells were co-cultured on tryptone broth agar. For these latter competitions, the target cells were derived from strain Bt36, which carries a deletion of the entire *cdiAIB* ^E264^ gene cluster [9]. CdiA-CT_II_^Bp1026b^ toxicity was tested by expressing the toxin domain inside *B*. *thailandensis* cells. Plasmid pSCBAD-CTII1026b was introduced into *E*. *coli* DH5α and the resulting strain used in a four-parent mating with SM10λpir/pTNS3 [23,24], HB101 (pRK2013) [25] and *B*. *thailandensis* E264. Conjugation mixtures were split into two equal portions and plated on LB agar supplemented with PB, Tp and 0.2% D-glucose and LB agar supplemented with PB, Tp and 0.2% L-arabinose. The presence of exconjugants on plates supplemented with D-glucose and the simultaneous absence of exconjugant colonies after incubation at 37°C for 48 h on the L-arabinose containing plates was indicative of toxicity.

**Table 1:** Bacterial strains used in this study.

Strains	Description	Source or Reference
*B*. *thailandensis* E264	wild-type isolate	[54]
Bt5	*B*. *thailandensis* E264 (pJSW2)	This study
Bt6	*glmS1*::Tn7-Kan, Kan^R^	[9]
Bt7	*glmS1*::Tn7-*cdiI* ^1026b^-Kan, Kan^R^	[9]
Bt28	BTH_II0599::T23(IS*lacZ*-P*rhaB*o-FRT-Tp); T23 transposon inserted after nucleotide 557 of coding sequence, Tp^R^	This study
Bt29	BTH_II0599::T23(IS*lacZ*-P*rhaB*o-FRT-Tp); T23 transposon inserted after nucleotide 611 of coding sequence, Tp^R^	This study
Bt30	BTH_II0599::T23(IS*lacZ*-P*rhaB*o-FRT-Tp); T23 transposon inserted after nucleotide 720 of coding sequence, Tp^R^	This study
Bt32	BTH_I0359::T23(IS*lacZ*-P*rhaB*o-FRT-Tp); T23 transposon inserted after nucleotide 226 of coding sequence, Tp^R^	This study
Bt33	BTH_I0986::T23(IS*lacZ*-P*rhaB*o-FRT-Tp); T23 transposon inserted after nucleotide 514 of coding sequence, Tp^R^	This study
Bt35	BTH_II0599::T23(IS*lacZ*-P*rhaB*o-FRT-Tp); T23 transposon inserted after nucleotide 524 of coding sequence, Tp^R^	This study
Bt36	Δ*cdiAIB glmS1*::Tn*7-kan*, Kan^R^	[9]
Bt45	BTH_II0599::T23(IS*lacZ*-P*rhaB*o-FRT-Tp); T23 transposon inserted after nucleotide 664 of coding sequence, Tp^R^	This study
Bt47	BTH_I0359::T23(IS*lacZ*-P*rhaB*o-FRT-Tp); T23 transposon inserted after nucleotide 49 of coding sequence, Tp^R^	This study
Bt49	BTH_I0986::T23(IS*lacZ*-P*rhaB*o-FRT-Tp); T23 transposon inserted after nucleotide 207 of coding sequence, Tp^R^	This study
Bt50	BTH_II0599::T23(IS*lacZ*-P*rhaB*o-FRT-Tp); T23 transposon inserted after nucleotide 371 of coding sequence, Tp^R^	This study
Bt51	BTH_II0599::T23(IS*lacZ*-P*rhaB*o-FRT-Tp); T23 transposon inserted after nucleotide 521 of coding sequence, Tp^R^	This study
Bt56	Δ*cdiAIB glmS1*::Tn7-P_*rpsL*_-*cdiI* ^E264^-*kan*, Kan^R^	[9]
Bt79	*glmS1*::Tn7-P_*rpsL*_-*gfp-kan*, Kan^R^	T. Hoang
Bt81	pJSW1–6, Tet^R^	[9]
Bt83	Δ*cdiAIB glmS1*::Tn*7-kan* ΔBTH_I0986, Kan^R^	This study
Bt87	*glmS1*::Tn*7-kan* ΔBTH_II0599, Kan^R^	This study
Bt101	*glmS1*::Tn*7-kan* pSCBAD::DsRed, Kan^R^ Tp^R^	This study
Bt103	*glmS1*::Tn*7-kan* ΔBTH_I0986, Kan^R^	This study
Bt104	Δ*cdiAIB glmS1*::Tn*7-kan* ΔBTH_II0599, Kan^R^	This study
Bt111	*glmS1*::Tn*7-kan* ΔBTH_I0986 pSCBAD::I0986, Kan^R^ Tp^R^	This study
Bt121	*glmS1*::Tn*7*-P_*rpsL*_-*gfp-kan* pJSW1–6, Kan^R^ Tet^R^	This study
Bt123	*glmS1*::Tn*7-kan* ΔBTH_I0986 pSCBAD::DsRed, Kan^R^ Tp^R^	This study
Bt124	*glmS1*::Tn*7-kan* ΔBTH_II0599 pSCBAD::DsRed, Kan^R^ Tp^R^	This study
Bt132	*glmS1*::Tn*7-kan* ΔBTH_I0359, Kan^R^	This study
Bt134	Δ*cdiAIB glmS1*::Tn*7-kan* ΔBTH_I0359, Kan^R^	This study
Bt137	*glmS1*::Tn*7-kan* ΔBTH_II0599 pSCBAD::II0599, Kan^R^ Tp^R^	This study
Bt138	*glmS1*::Tn*7-kan* ΔBTH_I0359 pSCBAD::I0359, Kan^R^ Tp^R^	This study
Bt143	*glmS1*::Tn*7-kan* ΔBTH_I0359 pSCBAD::DsRed, Kan^R^ Tp^R^	This study

Abbreviations: Kan^R^, kanamycin-resistant; Tet^R^, tetracycline-resistant; Tp^R^, trimethoprim-resistant

**Table 2:** Plasmids used in this study.

Plasmid	Description	Source or Reference
pEX18-Tp	Suicide vector containing pheS* gene for *o*-chlorophenylalanine counter-selection, Tp^R^	[55]
pSCRhaB2	Rhamnose-inducible promoter, Tp^R^	[27]
pSCBAD	Derivative of pSCRhaB2 with *E*. *coli araC* and P_BAD_ promoter, Tp^R^	This study
pSCBAD-KX	Derivative of pSCRhaB2 with *E*. *coli araC* and P_BAD_ promoter, Tp^R^	This study
pJSW2	Shuttle vector carrying *oriVpVS1 oriVp15A oriT araC-*P_BAD_,Tet^R^	[9]
pJSW1–6	pJSW2-*cdiAIB*_II_^1026b^, expresses the *Bp* 1026b CDI_II_ system under control of the arabinose-inducible P_BAD_ promoter, Tet^R^	[9]
pEX18-Tp:: ΔBTH_I0359	BTH_I0359 deletion construct, Tp^R^	This study
pEX18-Tp:: ΔBTH_II0599	BTH_I0599 deletion construct, Tp^R^	This study
pEX18-Tp:: ΔBTH_I0986	BTH_I0986 deletion construct, Tp^R^	This study
pSCBAD-KX::0359	Arabinose-inducible expression of BTH_I0359, Tp^R^	This study
pSCBAD::0599	Arabinose-inducible expression of BTH_I0599, Tp^R^	This study
pSCBAD::0986	Arabinose-inducible expression of BTH_I0986, Tp^R^	This study
pCH450-CT_II_^1026b^	Arabinose-inducible expression of residues Met2821—Asn3122 of CdiA_II_^Bp1026b^, Tet^R^	[9]
pSCBAD- CT_II_^1026b^	Arabinose-inducible expression of residues Met2821—Asn3122 of CdiA_II_^Bp1026b^, Tp^R^	This study
pTrc-DsRed	IPTG-inducible expression of DsRed, Amp^R^	[8]
pSCBAD::DsRed	Arabinose-inducible expression of DsRed, Tp^R^	This study

Abbreviations: Amp^R^, ampicillin-resistant; Tet^R^, tetracycline-resistant; Tp^R^, trimethoprim-resistant.

### Selection of CDI^R^ mutants

A library of random T23-Tp^R^ transposon-insertion mutants (>38,000 unique insertions) [26] was co-cultured with *B*. *thailandensis* Bt81 inhibitor cells [9]. Inhibitors and mutant target cells were mixed at a 10:1 ratio and plated onto M9 minimal agar medium supplemented with 0.2% L-arabinose 1 μg/mL thiamine and 0.3 μg/mL ferric citrate. After 24 h co-culture at 37°C, cells were harvested from the agar surface and surviving target cells isolated on Tp-supplemented LB agar. The target cells were pooled and subjected to two additional rounds of selection against CDI_II_^Bp1026b^ expressing inhibitor cells. After enrichment, individual clones were selected and tested for CDI-resistance in competition co-cultures with Bt81 inhibitor cells. Transposon-insertion junctions were amplified by arbitrary PCR using primers LacZ-124L2, LacZ-148 and CEKG 2E/K/L (Table 3). The resulting products were sequenced with primers LacZ211 and CEKG4 to identify insertion sites (Table 3).

**Table 3:** Oligonucleotides used in this study.

Oligonucleotide	Sequence^*a*^	Reference
2725	5´—ATA Tcc cgg gTC ATC GAT CGG AGG TGT TCG	This study
2729	5´—ATA Tcc cgg gTC ATC GCC CTC CGT TAC G	This study
3103	5´—CAA CAA aag ctt CAT CGA CAC GCT CGT GGG AGA	This study
3104	5´—GAT CGT ACT GGA TCG CTGC ACG CCA AAA ACC AAC GGC CGG ACC C	This study
3105	5´—GCG TGC AGC GAT CCA GTA CGA TC	This study
3106	5´—CAA CAA ggt acc CGT GTC GCC GAG CAA CAG ATG A	This study
3182	5´—CAA CAA aag ctt CAT CAG CCG AAC CTG CGC AGC	This study
3183	5´—GAT CGG AGG TGT TCG GCA GCT TCG CGG AAC CAC ACG TAG CCG G	This study
3184	5´—GAA GCT GCC GAA CAC CTC CGA TC	This study
3185	5´—CAA CAA ggt acc GAG CAG CGG CTT GTA CGC CTT	This study
3258	5´—GCG Cga att cCG AGA CCC ACG CAT GCA AC	This study
3259	5´—GCG Cga att cCA GGG CGC CAT TCG ATG AC	This study
3296	5´—ATA Taa gct tCT GCG TGA TCG ACA AGA GC	This study
3297	5´—CCG CCA TGC AAA TGA TCT ACA ACC CGT CGT TCT CCA CTG	This study
3298	5´—CAG TGG AGA ACG ACG GGT TGT AGA TCA TTT GCA TGG CGG	This study
3299	5´—GCG Ctc tag aGA TCG GCG ACG AAA CGA TCT	This study
CH1730	5´—GTA cca tgg TAC CTT CCT CCT GCT AGC	This study
CH2059	5´—AGT ggt acc ATG CAA ATG ATC TAC AAC AGC	This study
CH2799	5´—GAT atg cat AAT GTG CCT GTC AAA TGG	This study
CH2800	5´—TAC TGC AGC CCT CGA GTC AGT GGA GAA CGA CG	This study
LacZ-124L2	5´—CAG TCA CGA CGT TGT AAA ACG ACG	This study
LacZ-148	5´—GGG TAA CGC CAG GTT TTT CC	This study
LacZ-211	5´—TGC GGG CCT CTT CGC TAT TA	This study
CEKG 2E	5´—GGC CAC GCT CGA CTA GTA CNN NNN NNN NNA TGT A	This study
CEKG 2K	5´—GGC CAC GCG TCG ACT AGT ACN NNN NNN NNN AGT GC	This study
CEKG 2L	5´—GGC CAC GCG TCG ACT ACN NNN NNN NNN CTG AG	This study
CEKG 4	5´—GGC CAC GCG TCG ACT AGT AC	This study

^*a*^Restriction endonuclease sites are in lowercase; N indicates equal mixture of all four deoxyribonucleotides.

## Construction of Plasmids and Chromosomal Deletions

Plasmid pSCBAD is a derivative of pSCRhaB2 [27]. The *araC* gene and *araBAD* promoter were excised from plasmid pCH450 [28] by NsiI/NcoI digestion and ligated to plasmid pSCRhaB2. This sub-cloning step replaces the original rhamnose-inducible promoter with an arabinose-inducible promoter. Plasmid pCH450 was amplified with primers CH1730/CH2799 and the resulting product cloned into pSCRhaB2 using NsiI/NcoI restriction sites to generate plasmid pSCBAD-KX. The BTH_II0599 and BTH_I0986 genes were amplified from chromosomal DNA using primers 3258/2725 and 3259/2729 (respectively), and the resulting products ligated to plasmid pSCBAD using EcoRI and XmaI restriction sites. BTH_I0359 was amplified using primers CH2059/CH2800 and ligated into pSCBAD-KX using KpnI and PstI restriction sites. The region encoding CdiA-CT_II_^Bp1026b^ (residues Met2821—Asn3122 of full-length CdiA_II_^Bp1026b^) was subcloned from plasmid pCH450-CT_II_^1026b^ [9] into pSCBAD using NcoI and PstI restriction sites. The DsRed coding sequence was subcloned from plasmid pTrc-DsRed [8] into pSCBAD using NcoI and PstI restriction sites.

Gene deletions were constructed by allelic exchange as described previously [29]. DNA sequences upstream and downstream of the target gene were amplified and the two PCR products combined into one fragment using overlapping end PCR (OE-PCR) [30]. The OE-PCR products were ligated to plasmid pEX18-Tp (Table 2) using HindIII and KpnI/XbaI restriction sites. The BTH_I0359 deletion construct was generated using primer pairs 3296/3297 and 3298/3299; the BTH_II0599 deletion construct was generated using primer pairs 3182/3183 and 3184/3185; and the BTH_I0986 deletion construct generated using primer pairs 3103/3104 and 3105/3106 (Table 3).

### Cell-cell adhesion

GFP-labeled *B*. *thailandensis* E264 [24] carrying plasmid pJSW1–6 (Bt121) or pJSW2 (Bt5) were grown overnight in tryptone broth, then diluted 1:50 in fresh tryptone broth and grown to OD_600_ ~0.5. DsRed-labeled target strains (Bt101, Bt123, Bt124 and Bt143) were grown in minimal M9-media supplemented with 0.2% L-arabinose for at least 48 h to OD_600_ ~ 0.5. Inhibitor and target cells were mixed at a 1:1 ratio and incubated for 30 min at room temperature to allow cell-cell binding. Cell suspensions were then diluted 1:50 into sterile filtered 1X phosphate buffered saline and analyzed by flow cytometry. Samples were run on an Accuri C6 flow cytometer using FL1 (533/30nm, GFP) and FL2 (585/40nm, DsRed) fluorophore filters. Cell-cell binding was measured as the percent of target cells in aggregates with inhibitor cells divided by the total number of target cells. Binding data were normalized to the level of cell-cell binding between wild-type target cells (Bt101) and CDI_II_^Bp1026b^ expressing inhibitors (Bt121).

### Lipopolysaccharide (LPS) analysis

Bacteria were grown to OD_600_ > 0.6 in M9-minimal medium supplemented with 0.2% L-arabinose and LPS was harvested from an equivalent of 10 mL of OD_600_ = 1 culture using the LPS Extraction Kit (Boca Scientific, USA). Purified LPS was resolved on a 4–20% polyacrylamide Tris-glycine SDS gel (Thermo Scientific) and visualized using ProQ 300 Emerald LPS stain (Molecular Probes, USA).

## Results

### Isolation of CDI^R^ mutants

**Fig 1:**
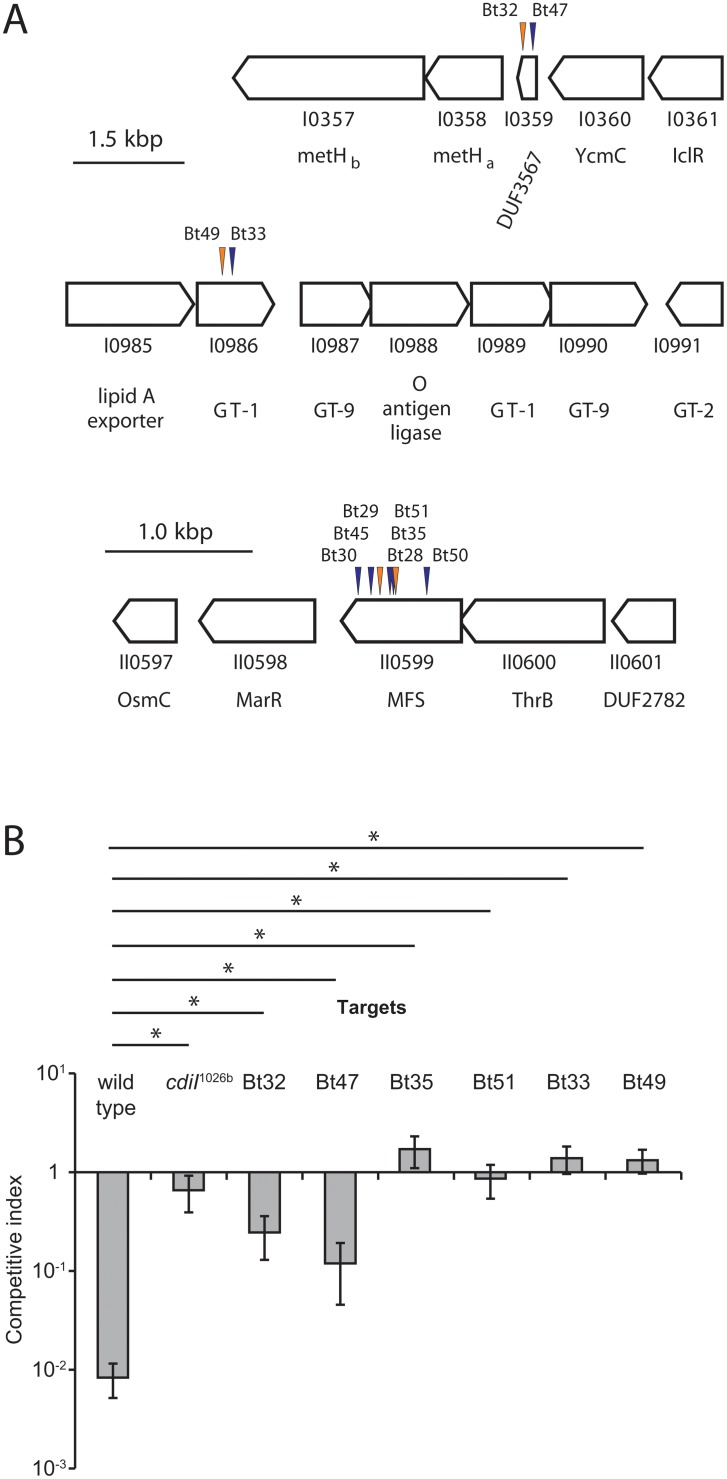
Selection of CDI^R^ mutants of *B*. *thailandensis* E264. A) T23 transposon insertion sites were identified by semi-arbitrary PCR as described in Methods. Orange arrows indicate T23 insertions in the same transcriptional orientation of the disrupted gene and blue arrows indicate insertions in the opposite orientation. The corresponding CDI^R^ mutant strain number is given above each arrow. Automated gene annotations are given below each ordered locus designation. GT-1, GT-2 and GT-9 indicate predicted glycosyltransferase families and DUF designations indicate domains of unknown function. B) The indicated *B*. *thailandensis* strains were co-cultured with Bt81 inhibitors (Table 1) that express the CDI_II_^Bp1026b^ system for 24 h on solid medium, and the competitive index was calculated as described in Materials and Methods. The strain labeled *cdiI* ^1026b^ expresses the cognate CdiI_II_^Bp1026b^ immunity protein. Data represent the mean ± SEM for three independent experiments. Analysis of the data using Student’s *t-*test is shown at the top, with bars between samples that were statistically significant (* = p< 0.05).

To gain insight into the CDI pathways in *Burkholderia* species, we used a genetic approach to identify target-cell genes that are required for growth inhibition. We reasoned that mutants with disruptions in the genes encoding the CDI receptor, toxin translocators and toxin activators would be CDI-resistant (CDI^R^). *B*. *thailandensis* E264 cells were subjected to random mutagenesis using a Tn*5*-based T23 transposon. Two independent T23 mutant pools were then co-cultured on solid media with *B*. *thailandensis* inhibitor cells that express the *B*. *pseudomallei* CDI_II_^Bp1026^ system from a plasmid vector (Bt81, Table 1). CDI^R^ mutants were enriched through three cycles of co-culture with inhibitor bacteria, and 20 clones were selected for the identification of transposon insertion sites. Each mutant contained a T23 insertion within BTH_I0359, BTH_II0599 or BTH_I0986; corresponding to eleven unique insertion sites (Fig. 1A and Table 1). BTH_I0359 is located upstream of the genes for methionine biosynthesis and encodes a hypothetical protein of 85 amino acid residues (Fig. 1A). BTH_II0599 encodes a predicted major facilitator superfamily (MFS) protein and is likely to be an inner-membrane localized transporter. BTH_I0986 is annotated as lipooligosaccharide (LOS) glycosyl-transferase G and is located within an LPS biosynthesis operon on chromosome I (Fig. 1A). We picked two mutants for each disrupted gene and confirmed that each was 10- to 100-fold more resistant to the CDI_II_^1026b^ system than wild-type *B*. *thailandensis* (Fig. 1B).

**Fig 2:**
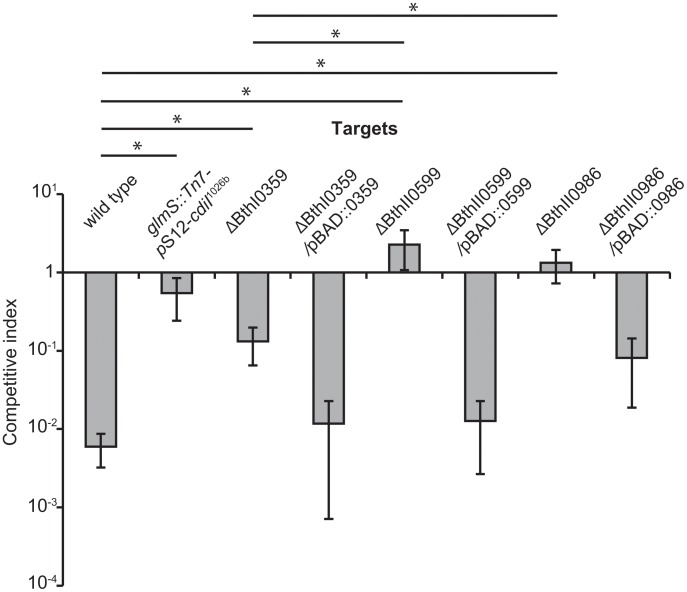
Complementation of CDI^R^ mutations. The indicated *B*. *thailandensis* strains were co-cultured with Bt81 inhibitors (Table 1) that express the CDI_II_^Bp1026b^ system for 24 h on solid medium, and the competitive index was calculated as described in Materials and Methods. The strain labeled *cdiI* ^1026b^ expresses the cognate CdiI_II_^Bp1026b^ immunity protein. Plasmid-borne copies of BTH_I0359, BTH_I0986 and BTH_II0599 genes were expressed from an L-arabinose inducible promoter. Data represent the mean ± SEM for three independent experiments. Sample values that were statistically different from one another (p < 0.05) are shown by bars with an asterisk (see Fig. 1).

Because multiple independent insertions were identified for each gene, it is likely that these mutations are directly responsible for the CDI^R^ phenotype. However, it is possible that the mutant strains carry additional unidentified mutations that contribute to resistance. To ascertain the roles of BTH_I0359, BTH_I0986 and BTH_II0599 in CDI, we constructed in-frame deletions of each gene and tested the resulting mutants for CDI^R^. As expected, the deletion mutants each had CDI^R^ phenotypes that were very similar to the originally isolated transposon-insertion mutants (Figs. 1B and 2). ΔBTH_I0986 and ΔBTH_II0599 mutants were fully resistant to CDI_II_^Bp1026b^, whereas the ΔBTH_I0359 mutant was only partially protected from inhibition (Fig. 2). These results strongly suggest that each gene is required for the CDI_II_^Bp1026b^ inhibition pathway. We also showed that each deletion mutant was rendered sensitive to CDI_II_^Bp1026b^ when complemented with a plasmid-borne copy of the appropriate gene (Fig. 2). These latter data exclude effects from transcriptional polarity and indicate that BTH_I0359, BTH_II0599 and BTH_I0986 are required for full sensitivity to the CDI_II_^Bp1026b^ system.

### Resistance mutations are specific for the CDI_II_^Bp1026b^ system

**Fig 3:**
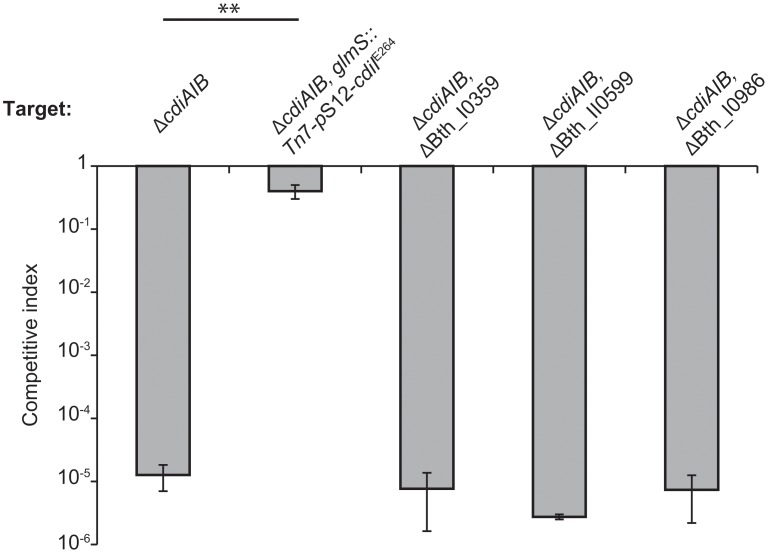
The CDI^R^ phenotype is specific for CDIII^Bp1026b^. The indicated *B*. *thailandensis* strains were co-cultured with wild-type (*cdiAIB* ^+^) *B*. *thailandensis* E264 cells for 24 h on solid medium, and the competitive index was calculated as described in Materials and Methods The strain labeled *cdiI* ^E264^ expresses the cognate CdiI^E264^ immunity protein. Data represent the mean ± SEM for three independent experiments. Sample values that were statistically different from one another (p < 0.01) are shown by a bar with a double asterisk (see Fig. 1).

*B*. *thailandensis* E264 carries its own CDI system (CDI^E264^) and the CdiA^E264^ protein shares approximately 53% sequence identity with CdiA_II_^Bp1026b^. However, the CdiA-CT^E264^ and CdiA-CT_II_^Bp1026b^ toxins are not homologous and have different nuclease activities [9], suggesting that the two toxin-delivery pathways could be distinct. Therefore, we asked whether mutations in BTH_I0359, BTH_II0599 and BTH_I0986 also provide resistance to CDI^E264^. We first confirmed that Δ*cdiAIB* ^E264^ mutants, which lack immunity to CDI^E264^, are inhibited by wild-type CDI^+^ *B*. *thailandensis* cells as reported previously [9,20]. *B*. *thailandensis* Δ*cdiAIB* ^E264^ targets were inhibited approximately 10^5^-fold during co-culture with CDI^+^ cells (Fig. 3). This growth inhibition is attributable to CDI^E264^, because the target cells were fully protected when complemented with the *cdiI* ^E264^ gene on a Tn*7*-based vector (Fig. 3). We then introduced ΔBTH_I0359, ΔBTH_II0599 and ΔBTH_I0986 mutations into the Δ*cdiAIB* ^E264^ background and found that each of the resulting strains was still sensitive to CDI^E264^ (Fig. 3). These results demonstrate mutations in BTH_I0359, BTH_II0599 and BTH_I0986 specifically confer resistance to the CDI_II_^Bp1026b^ system.

### CDI^R^ genes are not required to activate the CdiA-CT_II_^Bp1026b^ toxin

**Fig 4:**
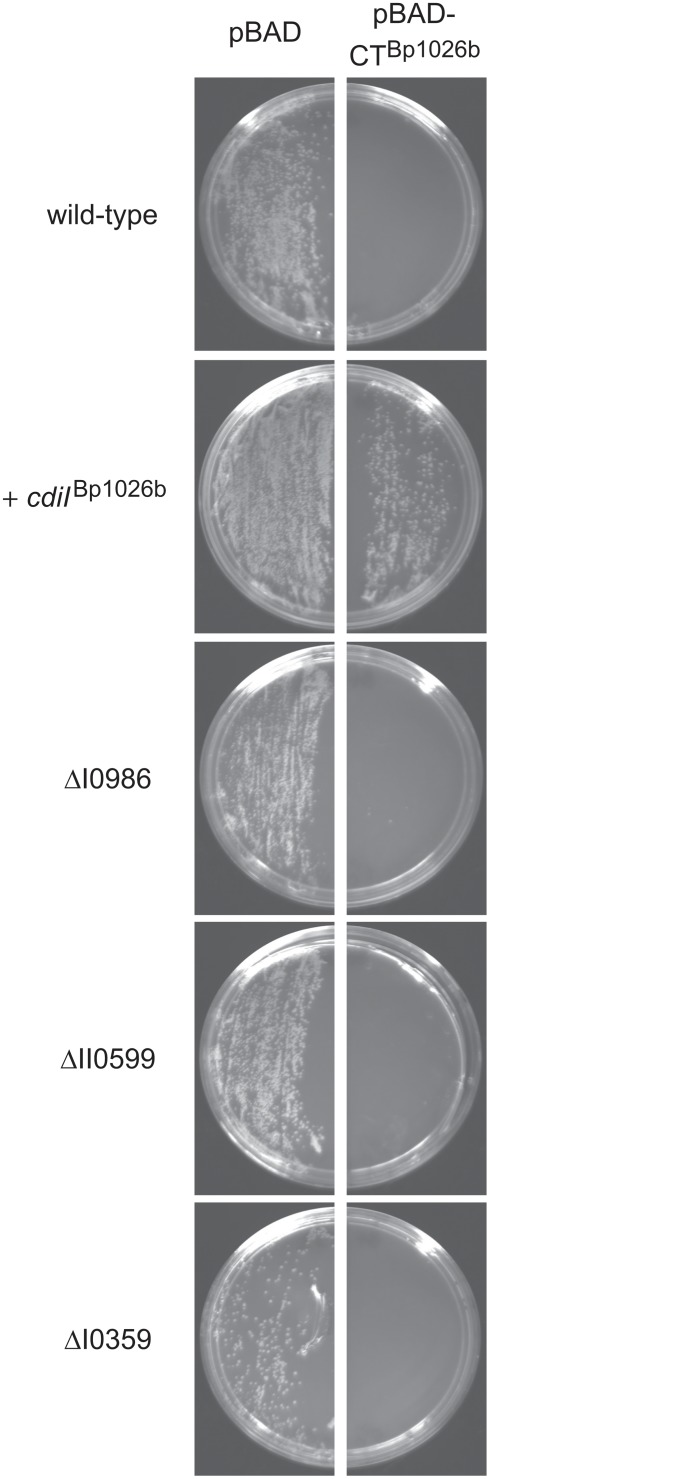
Toxicity of CdiA-CTII^Bp1026b^ expressed inside *B*. *thailandensis* cells. Plasmids pSCBAD and pSCBAD::*cdiA-CT*_II_^Bp1026b^ were introduced into the indicated *B*. *thailandensis* strains by conjugation as described in Materials and Methods. The mating mixtures were split into equal portions and plated onto LB agar with Polymyxin B and Trimethoprim supplemented with either D-glucose (left panels) or L-arabinose (right panels). See Materials and Methods.

Work with the CDI^536^ system from UPEC 536 has shown that some CDI toxins must be activated by so-called "permissive" factors. CdiA-CT^536^ only has tRNase activity when bound to CysK, and therefore *E*. *coli* Δ*cysK* mutants are completely resistant to the toxin, even when produced at high levels inside the cell [13,31]. Based on the CDI^536^ paradigm, we asked whether any of the *Burkholderia* CDI^R^ genes encode proteins with permissive factor function. We placed the *cdiA-CT*_II_^Bp1026b^ coding sequence under control of an arabinose-inducible P_BAD_ promoter and moved the construct onto a mobilizable plasmid. This plasmid can be stably maintained in *E*. *coli* cells under conditions that repress transcription from P_BAD_ [9]. We then tested whether the *cdiA-CT*_II_^Bp1026b^ plasmid could be introduced into *B*. *thailandensis* cells through tri-parental mating. No exconjugants were produced from matings to introduce the toxin plasmid into wild-type cells, but dozens of exconjugants were obtained when recipient cells expressed the cognate *cdiI*_II_^Bp1026b^ immunity gene (Fig. 4). These results indicate that CdiA-CT_II_^Bp1026b^ is toxic when expressed inside *B*. *thailandensis* cells and that CdiI_II_^Bp1026b^ neutralizes the toxin to allow cell growth. We next performed matings with ΔBTH_I0359, ΔBTH_II0599 and ΔBTH_I0986 recipient strains, each of which produced no exconjugants (Fig. 4). Together, these results show that none of the CDI^R^ mutations protect the cell from intracellular CdiA-CT_II_^Bp1026b^, indicating that the corresponding gene products do not function as CDI permissive factors.

### BTH_I0986 influences the binding of inhibitor and target cells

**Fig 5:**
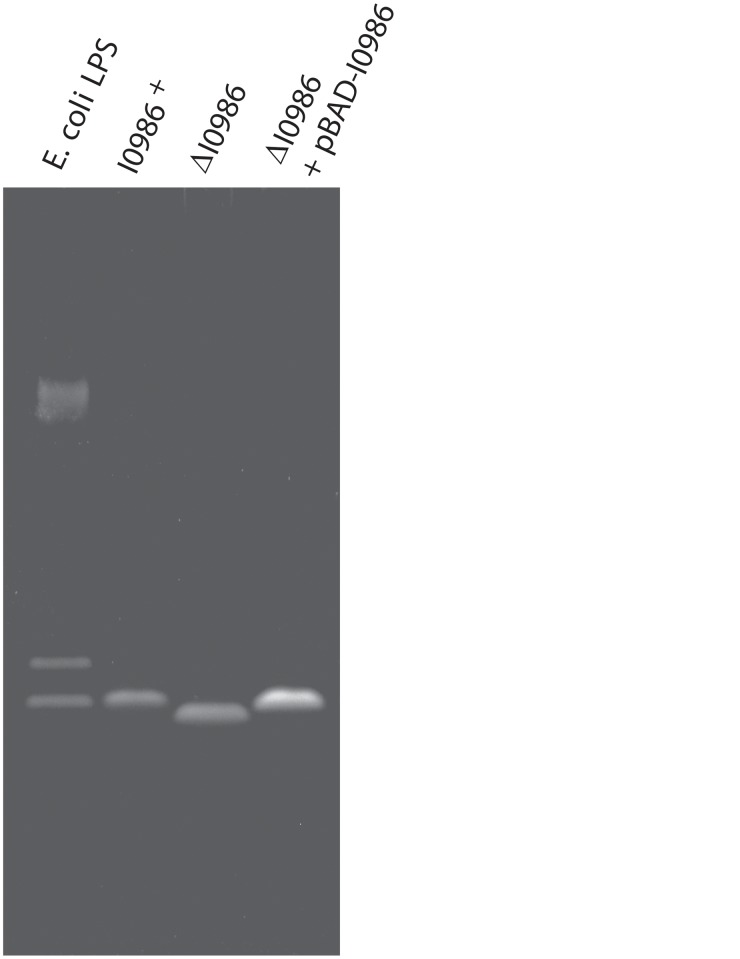
Lipopolysaccharide (LPS) analysis. LPS was isolated from the indicated *B*. *thailandensis* strains and analyzed by SDS-PAGE using fluorescent detection. The LPS standard is from *Escherichia coli* serotype 055:B5.

We next considered the possibility that the CDI^R^ genes may influence the recognition of target cells. The BTH_I0986 mutation is of particular interest because this gene belongs to the GT1 family of glycosyltransferases and is predicted to function in lipopolysaccharide (LPS) biosynthesis. Thus, the BTH_I0986 mutation could alter LPS structure, thereby preventing CDI_II_^Bp1026b^ inhibitor cells from recognizing and/or binding to target bacteria. To determine whether BTH_I0986 influences LPS structure, we used SDS-PAGE to analyze LPS isolated from wild-type and ΔBTH_I0986 cells. Surprisingly, we found that LPS isolated from wild-type *B*. *thailandensis* E264 cells lacked polymeric O antigen (Fig. 5), in contrast to previous reports [32,33]. The LPS from ΔBTH_I0986 mutants also lacked an O-antigen ladder, but migrated more rapidly during electrophoresis than LPS from BTH_I0986^+^ cells (Fig. 5). Complementation with plasmid-borne BTH_I0968 restored mutant LPS to the wild-type mobility (Fig. 5). Therefore, disruption of BTH_I0986 alters the target-cell surface by changing LPS structure.

**Fig 6:**
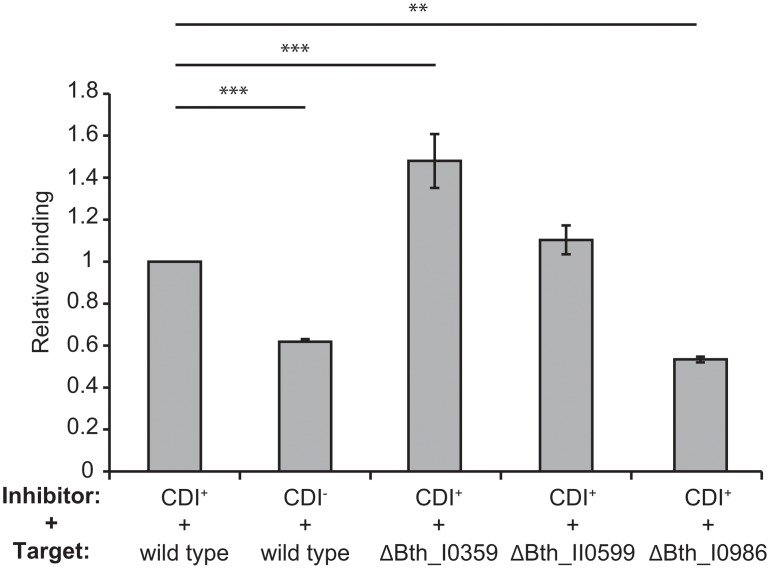
Cell-cell binding. CDI^+^ (Bt81) and CDI^–^ (wild-type *B*. *thailandensis*) cells were labeled with GFP and mixed with the indicated DsRed-labeled target cells, then analyzed by flow cytometry to detect and quantify cell-cell aggregates. Binding was normalized to 1.0 for the interaction between Bt81 and wild-type *B*. *thailandensis* cells. Sample values that were statistically different from one another are shown by bars; ** = p < 0.01, and *** = p < 0.001 (see Fig. 1). We then tested the three CDI^R^ target strains and found that ΔBTH_I0986 targets interacted poorly with inhibitor cells, similar to the level observed with CDI^–^ mock inhibitors (Fig. 6). In contrast, the ΔBTH_II0599 mutant showed wild-type binding levels, and ΔBTH_I0359 targets showed increased binding to inhibitor cells (Fig. 6). Together, these results suggest that mutations in BTH_I0986 confer CDI^R^ by altering the cell surface to prevent stable associations with CDI_II_^Bp1026b^ inhibitor cells.

In the *E*. *coli* EC93 system, inhibitor cells bind stably to target bacteria and the resulting cell aggregates can be detected and quantified using flow cytometry [12,18]. Therefore, we used the same approach to examine the binding of CDI_II_^Bp1026b^ inhibitors to different target cell strains. We mixed GFP-labeled CDI_II_^Bp1026b^ inhibitors at a 1:1 ratio with DsRed-labeled target cells and analyzed the suspensions by flow cytometry to detect events with both green and red fluorescence, which correspond to aggregates containing both inhibitor and target cells. This analysis showed that approximately 40% more target cells bind to CDI_II_^Bp1026b^ inhibitors compared to CDI^–^ mock inhibitors (Fig. 6).

## Discussion

**Fig 7:**
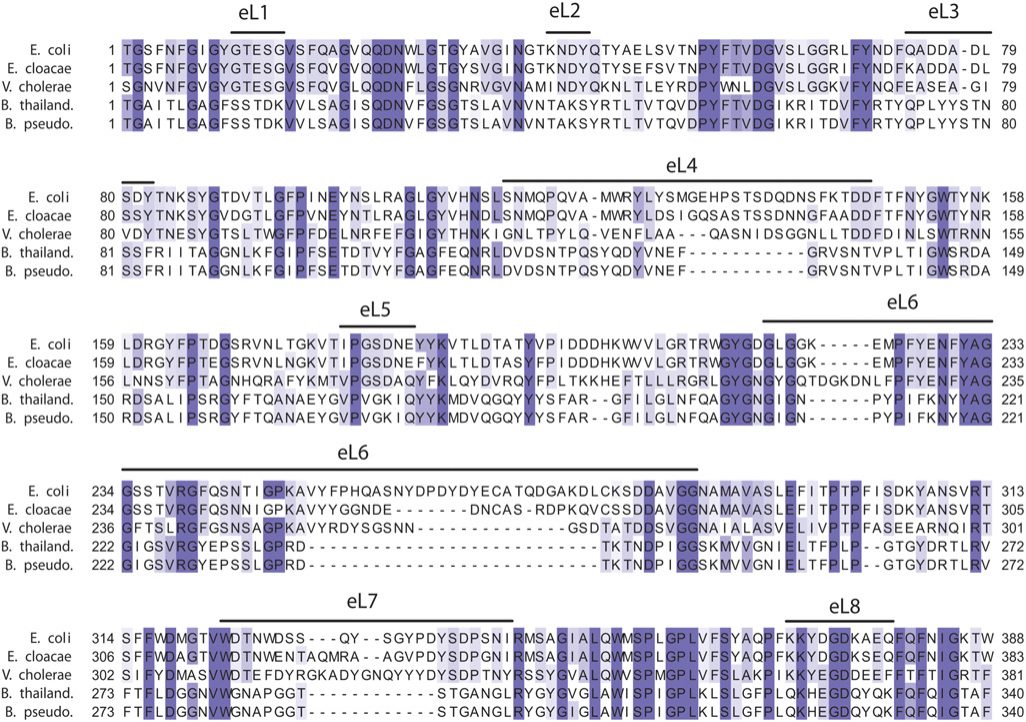
Alignment of BamA proteins. The β-barrel portion of BamA proteins from *E*. *coli* K-12 (Uniprot: P0A940), *Enterobacter cloacae* ATCC 13047 (D5CHY0), *Vibrio cholerae* ATCC 39315 (Q9KPW0), *B*. *thailandensis* E264 (Q2SWZ0) and *B*. *pseudomallei* 1026b (I1WHZ2). Sequences that correspond to extracellular loops (eL) are indicated above the alignment and are based on the crystal structures of BamA from *Neisseria gonorrhoeae and Haemophilus ducreyi* [37]. The alignment was rendered using Jalview 2.8 [53] at 30% sequence identity.

The results presented here show that at least three genes, BTH_I0359, BTH_I0986 and BTH_II0599, are required for *B*. *thailandensis* cells to be fully inhibited by the CDI_II_^Bp1026b^ system. We identified each gene in two independent selection experiments, suggesting that they represent the major non-essential genes required for the CDI_II_^Bp1026b^ pathway. Indeed, BTH_II0599 and BTH_I0986 are particularly critical because deletion of either gene provides full resistance to target bacteria. Notably, the three *B*. *thailandensis* genes identified here are distinct from those previously identified in *E*. *coli* within the CDI^EC93^ growth inhibition pathway [12]. These results suggest that the CDI_II_^Bp1026b^ and CDI^EC93^ systems deliver toxins through different pathways. CDI is initiated through direct binding interactions between CdiA and receptors on the surface of target bacteria. CdiA^EC93^ uses the *E*. *coli* BamA protein as a receptor and appears to bind specific epitopes within extracellular loops eL6 and eL7 [12,18]. Our results here suggest that *B*. *pseudomallei* CdiA_II_^Bp1026b^ may exploit LPS as a target-cell receptor. BTH_I0986 is a predicted transglycosylase and mutants lacking this enzyme have altered LPS structure (Fig. 5). Moreover, the ΔBTH_I0986 mutant shows defects in binding to CDI_II_^Bp1026b^ inhibitor cells (Fig. 6), consistent with a role in receptor function. Surprisingly, we also found that our *B*. *thailandensis* E264 isolate lacks a detectable O-antigen ladder. This could account for the fact that we did not identify any additional LPS biosynthesis genes in independent selections. It is unclear whether the rough LPS phenotype reflects phase variation [34–36], or whether a rough-strain mutant was selected through laboratory passage. In either event, it will be important to determine how O-antigen influences CDI susceptibility in *Burkholderia* species. Although our results do not support a role for BamA in *Burkholderia* CDI, we acknowledge that CDI^R^ alleles of the essential *bamA* gene would be difficult to isolate using a transposon mutagenesis approach. If *Burkholderia* BamA does function as a CDI receptor, then the interactions must be distinct from the CDI^EC93^ system, because BamA loops eL6 and eL7 loops differ significantly between *E*. *coli* and *Burkholderia* species (Fig. 7) [37].

Because CdiA-CT_II_^Bp1026b^ is a tRNase, this toxin must be transported into the target-cell cytoplasm to reach its substrate. CDI toxin translocation is poorly understood, but our recent work with *E*. *coli* indicates that transport across the target-cell outer membrane is energy-independent, whereas translocation into the cytoplasm requires the proton-motive force [38]. These findings raise the possibility that BTH_II0599, a predicted MFS transporter, is co-opted to translocate the tRNase domain across the target-cell inner membrane. In this model, periplasmic toxin would bind to BTH_II0599 and be driven into the cytoplasm by either the chemical or electrical potential of the pmf. These interactions are specific because the ΔBTH_II0599 mutation provides no protection against the *B*. *thailandensis* CDI^E264^ system, suggesting that the CdiA-CT^E264^ toxin must exploit another entry pathway. Although MFS proteins harness chemiosmotic gradients to transport a variety of metabolites [39,40], it seems unlikely that the transporter could translocate a folded nuclease domain in the same manner as small solutes. One possibility is that CdiA-CT_II_^Bp1026^ has an autonomous membrane translocation activity, but requires BTH_II0599 as a receptor to facilitate insertion into the inner membrane. This model is similar to that proposed by Kleanthous and colleagues for the translocation of colicin nuclease domains, some of which interact with phospholipids and form pores in membranes [41–43].

The role of BTH_I0359 in the CDI_II_^Bp1026b^ pathway remains enigmatic, in part because the function of this gene is unknown. BTH_I0359 encodes a DUF3567 family member (PF12091, http://pfam.xfam.org/family/PF12091), which is only found within the order Burkholderiales. The gene neighborhood of BTH_I0359 includes the downstream *metH*_a_ and *metH*_b_ (which encode a split methionine synthase) and an upstream DUF3108 family member. DUF3567 and DUF3108 genes are linked throughout all the Burkholderiales, whereas linkage to *metH* is limited to *Burkholderia*, *Ralstonia* and *Cupriavidus* species. DUF3108 genes encode outer-membrane β-barrel proteins with a characteristic YmcC fold (PDB: 3FZX). Although strong genetic linkage is often indicative of a functional relationship, we did not isolate BTH_I0360 mutations in our CDI^R^ selections, even though this gene is not essential for *B*. *thailandensis* growth [26]. We have also excluded a "permissive" factor function for BTH_I0359. Permissive factors are target-cell proteins that are required to activate CdiA-CT toxins in the target-cell cytoplasm [13]. This conclusion is also supported by previous studies showing that purified CdiA-CT_II_^Bp1026b^ has tRNase activity *in vitro*, and therefore does not require an additional factor for activation [8].

All *B*. *pseudomallei* strains contain at least one CDI system, and some isolates carry up to three loci [9]. Each system can be placed into one of 10 different toxin/immunity groups [9,20], suggesting that CDI mediates competition between different *B*. *pseudomallei* strains. Using *B*. *thailandensis* as a model, Cotter and colleagues have recently demonstrated that such competition does in fact occur in mixed-strain biofilms, and that CDI influences the composition of these communities [20,44]. Additionally, there are indications that *B*. *pseudomallei* and *B*. *thailandensis* do not co-inhabit the same environmental niches [45], again suggesting that anti-bacterial competition systems shape their environmental distributions. If *Burkholderia* species do in fact directly antagonize one another in the environment, then type VI secretion systems (T6SS) are more likely to effect this competition. *B*. *thailandensis* and *B*. *pseudomallei* strains all carry multiple T6SS, which have been shown to deploy toxins against both bacteria and eukaryotic targets [46–49]. Moreover, a given T6SS is capable of killing many different species of Gram-negative bacteria [50–52]. In contrast, CDI is a receptor-mediated process, and therefore variations in the cell-surface receptor epitopes restrict inhibition activity to a subset of bacteria [18]. In accord with this general model, data presented here show that CDI^E264^ is significantly more effective against *B*. *thailandensis* targets than CDI_II_^Bp1026b^. Together, these observations indicate that CDI is used primarily to differentiate sibling cells from other closely related bacteria.
